# Helicopter parenting through the lens of reddit: A text mining study

**DOI:** 10.1016/j.heliyon.2023.e20970

**Published:** 2023-10-17

**Authors:** C. Keerthigha, Smita Singh, Kai Qin Chan, Nerina Caltabiano

**Affiliations:** a School of Social and Health Sciences, James Cook University, Singapore; b College of Healthcare Sciences, James Cook University, Cairns, Australia

**Keywords:** Helicopter parenting, Social media data, Reddit, Text mining, Python, Natural language processing, Latent dirichlet allocation (LDA)

## Abstract

The study aimed to understand Reddit users’ experience with helicopter parenting through first-hand accounts. Text mining and natural language processing techniques were employed to extract data from the subreddit r/helicopterparents. A total of 713 original posts were processed from unstructured texts to tidy formats. Latent Dirichlet Allocation (LDA), a popular topic modeling method, was used to discover hidden themes within the corpus. The data revealed common environmental contexts of helicopter parenting (i.e., school, college, work, and home) and its implication on college decisions, privacy, and social relationships. These collectively suggested the importance of autonomy-supportive parenting and mindfulness interventions as viable solutions to the problems posed by helicopter parenting. In addition, findings lent support to past research that has identified more maternal than paternal models of helicopter parenting. Further research on the implications of the COVID-19 pandemic on helicopter parenting is warranted.

## Introduction

Social media is gaining ground in our everyday life. Online communication and sharing on these platforms (e.g., Facebook, Twitter, and Instagram) often provide information about our psychological characteristics, attitudes, and behaviours [1]. The availability of social media data provides researchers with opportunities to gain deeper insights into users' social behaviour in an uncontrolled setting [2]. Assessing big data thus allows the unobtrusive analysis of trends and patterns that are specific to a particular issue. The present study examines data from the social media platform Reddit. Reddit users (*N* = 430,000,000; [3]) subscribe to user-created discussion groups termed ‘subreddits’ that often reflect specific interests (e.g., r/baking and r/dogs) and experiences (e.g., r/depression and r/mindfulness). Reddit's anonymous user identity allows for free responses without inhibition [4]. This enables the collection of sensitive and meaningful information to create an accurate map of users' experience with helicopter parenting (HP) in the present study. Posts from the subreddit r/helicopterparents are analysed using computational linguistics and qualitative synthesis. It is anticipated that findings contribute to the ongoing research on the impact of HP among emerging adults.

## 1 Helicopter parenting

Parallel to helicopters that swoop in to rescue at the first sign of trouble, helicopter parents are characterized by their distinct style of parental overinvolvement and micro-management [[5], [6], [7]]. HP commonly referred as *overparenting*, describes parents who exhibit high involvement, control, and autonomy-limiting behaviours toward their child [8]. Behaviourally, these are expected to manifest in an increase in advice and other directive behaviours, an attempt to shield the child from negative outcomes, instrumental support, and a preoccupation with the child's happiness [7]. Examples of HP include problem-solving and decision-making for their children, and intervening in their affairs [[9], [10]]. The cause of HP is yet to be fully understood with recent studies suggesting parental anxiety and regret [11,12], culture [13]; [14], and parent-child gender combinations [[15], [16], [17]] as strong contributing factors to HP.

HP has been primarily studied in the emerging adult population, which refers to the phase of life between adolescence and adulthood (typically ages of 18–29; [18]. During this transitional period, individuals experience a growing need for autonomy, whereby their decisions are guided only by personal preferences [13,19]. However, HP tends to impede autonomy development. Emerging adults encounter a multitude of stressors, such as identity and relationship formation and those with helicopter parents may face even greater difficulties in this stressful climate.

Insights from advanced imaging technology have demonstrated the structural and functional changes of the brain during the transitional period of development [20] and how it relates to social cognition. Social cognition refers to the ability to infer and reason, which plays a critical role in the successful negotiation of complex social interactions and decisions [21]. Coherently, research has shown HP exacerbates poor social adjustment and greater levels of alienation from college peers [22]. Reports from emerging adults also indicate HP may foster a family and social environment that impairs their relationship with others [7].

There is a significant amount of evidence linking high levels of anxiety, depression, low life satisfaction, and poor psychological well-being to HP [6,7,23,[24], [25]]. Identifying and addressing the sources of distress among emerging adults has become paramount to practitioners, higher education institutions, and families [26]. As emerging adults increasingly take to sharing their distress on social media, a growing body of research focuses on such self-disclosures as an unobtrusive way to uncover the hidden or suppressed views of their adversities and how it affects them [27].

## 2 Mining social data

With the advent of social media, online communities have emerged to provide help, advice, and support for those who share similar experiences [28]. Perceived support in these communities plays a prominent role in improving mental health by reducing stress [29], increasing self-efficacy [30], and fostering positive behaviour changes [31]. Most online platforms allow their users to post anonymously, providing a sense of security to discuss their experiences without the fear of being stigmatized or discriminated against [[32], [33], [34]]. Notably, social media facilitates communication among its users and produces a colossal amount of social data [35].

Social data is mined through text mining, an artificial intelligence technology that attempts to extract meaningful information from unstructured textual data [36]. The data is automatically indexed in specific ways (e.g., via common schemas) to create models which explain patterns and trends. Researchers have used this technology to analyse large amounts of textual data in business [37], health science [36], and educational [38,39] domains. They have also leveraged data to investigate human behaviour and interaction [40]. These have collectively provided solutions to real-world problems such as the detection of depression [41], childhood sexual abuse [42], and suicidal ideation [43].

Recent text mining applications have allowed social scientists to observe natural online behaviour, gaining deeper insights into users’ real-time psychosocial characteristics [2]. Natural Language Processing (NLP), a subfield of Artificial Intelligence, is an emerging technology that uses machines to understand human languages [44]. The NLP process comprises the following steps: (a) text preprocessing, in which the dataset is cleaned by removing non-textual information (e.g., emojis, images, and HTML tags); (b) text representation, where the dataset is transformed into word vectors; (c) model training, during which algorithms are utilised to train a model (e.g., sentiment analysis, opinion mining, or topic modeling); and (d) model evaluation, where the model is evaluated to ensure it has optimal generalisability to other corpora [45].

The use of NLP in psychological studies requires the text corpus to be processed according to vocabulary [46]. An open-vocabulary approach does not rely on a priori word or category judgments [47,48]. Instead, it aims to identify distinct sets of linguistic features (e.g., words, n-grams, and topics) in the corpus. In contrast, a closed-vocabulary approach is based on theoretical and empirical evidence on linguistic features [49]. This typically involves using word dictionaries that match words with a target psychological variable (e.g., anxiety, disgust, and happiness). Taken together, NLP offers social science a novel approach to data analysis which, in turn, creates accurate maps of a phenomenon.

The complexity of HP [50] necessitates more nuanced and in-depth examination through various scholarly inquiries such as text mining publicly available social data. Furthermore, despite the vast amount of literature on the adverse impact of HP on emerging adults [13,[51], [52], [53]], more significant effort is warranted to understand their negative experiences through first-hand perspectives and words [14]. This eliminates the disparities between lived experiences and their accounts, thus allowing authentic communication of their problems.

## 3 The present study

Given that research on HP has heavily relied upon empirical research [14], the present study aims to understand Reddit users’ experience with HP using NLP techniques, particularly its open-vocabulary approach. In this paper, a social media platform that discusses user experiences with helicopter parents (r/helicopterparents) will be examined. This serves as an attempt to contribute to the ongoing efforts to amalgamate both the distinct fields of psychology and computational linguistics research. It is thus anticipated that this study will make novel, scholarly contributions to the existing literature on HP. Due to the exploratory nature of this study, a research question (RQ), rather than an a priori hypothesis, is employed.

RQ: What are Reddit users’ experiences with HP?

## 4 Method

### 4.1 Research design and participants

A Big Data approach was used to extract and analyse textual data from an online forum, Reddit. This study used secondary data thus, no participants were recruited. Reddit users are typically between the ages of 18 and 29 years [54], thus assumed emerging adults.

### 4.2 Ethical considerations

Ethical clearance for the study was granted by the Human Research Ethics Committee James Cook University, Australia (Ref. H8491). There are no formal guidelines in the National Statement on Ethical Conduct in Human Research [55] about Big Data approaches. Hence, the study adhered to the recommendations of [56,57] for best practices. The recommended ethical code of conduct and the corresponding explanation for compliance in the present study is presented in the Appendix.

### 4.3 Corpus

The corpus was based on *r/helicopterparents*, a subreddit that discusses user experiences with HP. Reddit (http://www.reddit.com), is a popular online social networking and news exchanging platform. Reddit is largely based on threaded conversations which are common communication patterns that effectively capture information on a particular topic [35]. Reddit has multiple publicly available subreddits which are topically focused sub-communities. In these subreddits, users bring up conversations by sharing their experiences while others can choose to respond to the thread. As of January 24, 2022, r/helicopterparents has been active for approximately seven years with 15,118 members.

### 4.4 Data acquisition

The corpus was constructed using Reddit's official Application Programming Interface (API; Reddit, 2021), called the Python Reddit API Wrapper (PRAW; [58]. An API is a software intermediary that enables data transmission between one application to another without compromising underlying implementation [59]. PRAW is designed to respect all of Reddit's API rules. Data extraction of posts from November 11, 2014 to January 24, 2022 was conducted in compliance with Reddit API Terms of Service [60]. The API was instructed to eliminate the usernames and titles of the posts while collecting texts for the corpus. Only textual data found in the body of the posts were extracted. Responses to the original posts were not included in the corpus as this study aimed to identify common experiences with helicopter parents rather than describe the conversations surrounding these situations. Therefore, a total of 713 original posts across all threads were retained.

### 4.5 Data pre-processing

The corpus was prepared for analysis using the Natural Language Toolkit (NLTK) [61] on Python 3.9.5. The NLTK provided a collection of text processing libraries such as lowercase conversion, tokenization, stop-word removal, and punctuation removal. Stemming was also applied to reduce words to their root form and group them by similarity. Web addresses and Unicode characters (e.g., symbols and emojis) were automatically removed. These collectively helped eliminate noise and correct inconsistencies in the data, transforming unstructured texts to tidy formats. The pre-processed data was used to produce basic corpus statistics (i.e., time series and n-gram analyses) and extract topics.

### 4.6 Feature extraction

The present study used latent Dirichlet allocation (LDA), an open-vocabulary feature extractor to study the corpus [62]. LDA uses the Bayesian probabilistic modeling method to extract a set of topics from a corpus of text. Specifically, LDA generates estimates of words associated with each topic (i.e., word-topic probabilities) and estimates of topics describing the document (i.e., document-topic probabilities; [63]. Inspecting the highest word-topic and document-topic probabilities allows the identification of the theme of each latent topic. Furthermore, the unsupervised learning algorithm of LDA reduces researcher bias in selecting keywords, generating topics, and ranking topic prevalence in the document [64].

### 4.7 Qualitative synthesis

**Fig. 1:**
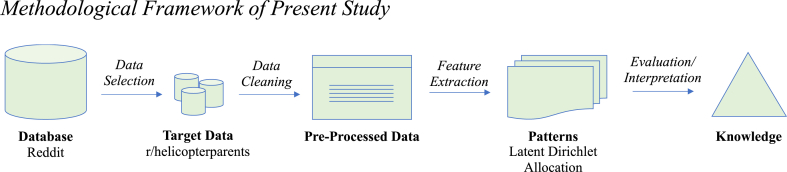
Methodological Framework of Present Study *Note.* The process consists of data selection, data cleaning, feature extractions, and evaluation and interpretation of results.

A qualitative approach was employed to contextualise the LDA model. This approach relied on the manual, in-depth interpretations of topics from authors, which allows for exploratory analysis of the findings. Each set of topical words was collectively assigned meanings, inductively developing themes for the latent topics generated by machine algorithms. Fig. 1 illustrates the methodological framework used in this study.

## 5 Results

### 5.1 Corpus statistics

**Fig. 2:**
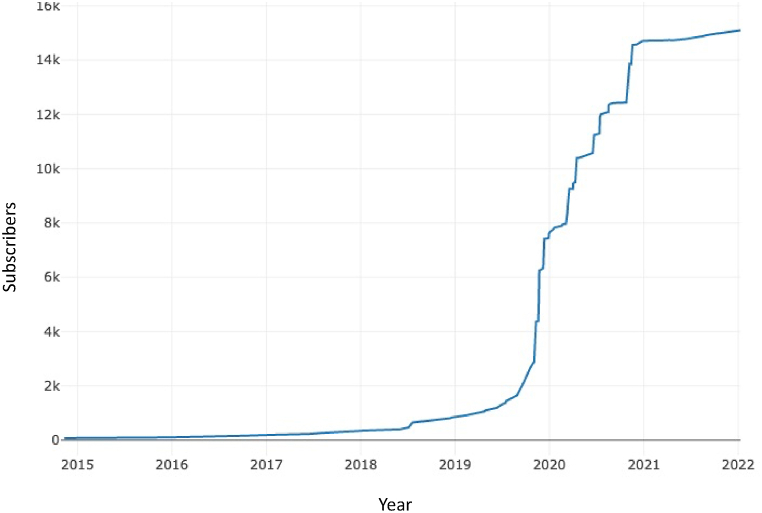
*Subscription Rate for the Subreddit r/helicopterparents* *Note.* A rapid increase in the number of subscribers is observed since late 2019.

A series of uptrends in the subscriber count were observed since late 2019 [65]. Fig. 2 illustrates the subscription rate for the subreddit r/helicopterparents.

### 5.2 Unigram analysis

**Fig. 3:**
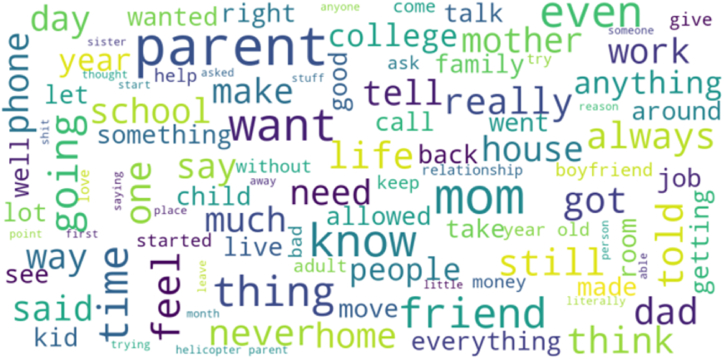
Visualization of unigram frequency.

The top 100 most frequently occurring unigrams are presented in a word cloud (see Fig. 3). The more prominent a word appears in the corpus, the bigger its representation in the word cloud. Findings consistently revealed the terms ‘mother’ and ‘mom’ as the most frequently used word in the data.

### 5.3 LDA

#### 5.3.1 Hyperparameter tuning

**Fig. 4:**
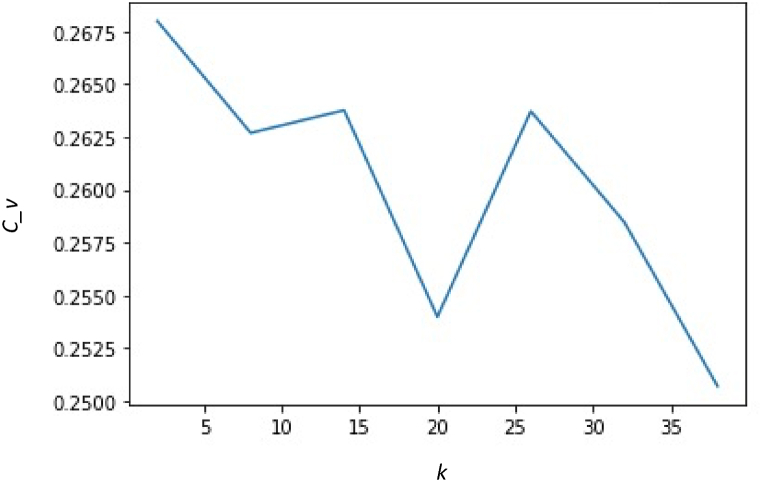
Determining the Optimal Number of Topics based on Topic Coherence Score *Note.* The optimal number of topics (k) vs coherence score (C_v) is presented.

Topic coherence measures the degree of semantic similarity between high-scoring words in a dataset [66] which is essential for human interpretation of topic models. Therefore, Gensim's *coherencemodel* was imported to evaluate topic models [67]. Fig. 4 outlines the optimal number of topics against the coherence score with a fixed alpha of .01 and beta of 0.1. The maximum coherence score of 0.26 was achieved when the number of topics was set to four.

### 5.4 Topic model visualization

**Fig. 5:**
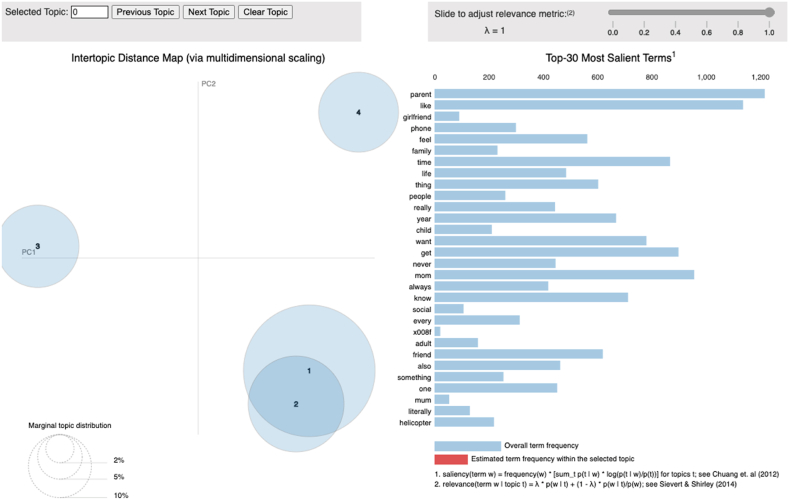
*Topic Model Visualization Using* pyLDAVis *in Gensim with four Topics* *Note.* The bubbles illustrate overall topic distribution while the bars represent overall word frequencies.

Gensim's *pyLDAVis* was imported to produce an interactive visualization of topics [68]. Fig. 5 shows the topic model visualization of the present study. While each bubble represents a topic, the more prevalent a topic is, the bigger its representation. Overlapping bubbles indicate similarities while the words listed on the right panel represent the top 30 most salient keywords found in the dataset.

Each topic is represented by its top 30 most salient and frequent keywords. Table 1 summarises the most representative words correlated with each topic as generated from the dataset and its corresponding themes.

**Table 1:** Summary of topical words and corresponding themes.

Topic No.	LDA Topical Words	Topic Themes	Description of Themes
1	*mom, time, friend, school, home, house, life, dad, college, work*	Environmental Contexts	School, college, work, and home are environemental contexts in which users are exposed to HP behaviours the most.
2	*time, mom, friend, life, home, school, room, mother*	College Decisions	Users' choice of friendship, school, accommodation, and lifestyle are limited by helicopter parents.
3	*time, life, friend, mom, school, people, child, phone, family*	Invasion of Privacy	Helicopter parents closely monitor users' activities, both online and offline.
4	*mom, time, friend, phone, home, family, dad, girlfriend*	Social Relationships	HP interferes with users' social lives with family members and romantic partners.

*Note.* Four unique but broadly defined experiences that users face with helicopter parents were identified through manual interpretation of the topical words.

## 6 Discussion

This paper sought to understand Reddit user experience with HP by identifying common experiences shared by users with helicopter parents. The key findings revealed four main domains, including the common environmental contexts of HP (i.e., school, college, work, and home) and its implication on college decisions, privacy, and social relationships. The following sections will first discuss these specific impacts of HP and its implications for emerging adults. Next, highlight the study's overall contributions, limitations, and directions for future research.

### 6.1 Environmental contexts

The data suggest that places such as school, college, work, and home are environmental contexts in which Reddit users are exposed to HP behaviours the most. This is in line with research that indicates helicopter parents readily intervene in their children's affairs with peers, professors, and employers [[9], [10]]. Given that autonomy is a key characteristic of the emerging adulthood developmental period [18], parents who impose excessive restrictions may imply a lack of faith in their children's ability to navigate situations independently. An autonomy-restrictive parenting style, such as HP, may negatively impact an individual's psychological well-being and ability to adjust in college or work settings [69]. Therefore, it is crucial to provide developmentally appropriate levels of autonomy suitable to their children's age.

### 6.2 Loss of control over college decisions

The data suggest that HP poses control over users’ college decisions and this is manifested in terms of a lack of control over peer relationships, school, accommodation, and lifestyle choices. This is consistent with past findings that suggest HP is related to a host of negative outcomes in college, including poor academic achievement, self-esteem, life satisfaction, and peer relationships [9,22,24,50,70,71]. For example, peer relationships are central to emerging adulthood as they offer significant provisions such as support, security, and companionship in times of stress [72]. The quality of peer relationships has been linked to self-esteem, prosocial behaviour, life satisfaction, and scholastic competence [22]. This suggests that peer relationships play a pivotal role in remedying the ill effects of HP. It is thus proposed that colleges actively create opportunities for students to connect with peers and assist students in the arduous task of establishing healthy boundaries with helicopter parents by encouraging individual decision-making.

### 6.3 Invasion of privacy

The data suggest that helicopter parents supervise users both online (e.g., usage of the phone) and offline (e.g., people they meet), thus directly invading privacy. This is in line with previous research that highlights the association between HP and excessive parental monitoring [73]. HP stunts youth disclosure and parental knowledge [74], which may in turn lead to youth relational aggression [75] and internalising problems [76]. Although HP may have legal ramifications related to privacy rights [77], research on privacy management by helicopter parents remains largely unexplored [78]. found that open communication among family members predicted the likelihood of emerging adults revealing private information to their parents. This suggests that privacy invasion is counterproductive to parents’ efforts to remain knowledgeable about their children. Taken together, findings discourage excessive parental monitoring while encouraging enhanced family communication patterns (e.g., adopting autonomy-supportive parental monitoring strategies [79] and negotiations on privacy.

### 6.4 Impaired social relationships

The data suggest that HP interferes with users' social lives with family members and romantic partners. Research has shown that HP is strongly associated with emerging adults’ insecure attachment to romantic partners [80] and stronger beliefs that being single is more advantageous than being married [25]. Overparented emerging adults generalise their low self-efficacy to their dating incompetence [81]. Moreover, HP may underlie attachment insecurity given that helicopter parents have constantly fed their children with attention, approvals, and reassurances which may not be received in similar amounts from their romantic partners. That is, helicopter parents are inadvertently promoting long-term singlehood while preventing their children from having negative experiences [25]. Therefore, it is recommended that helicopter parents consider autonomy-supportive parenting, which, in turn, may facilitate healthy relationship formation and maintenance.

## 7 Summary of implications and contributions

### 7.1 Reddit as an inexpensive source for high-quality data

This study is among the first to elucidate the substantital potential of social data in understanding parenting outcomes. Empirical research have been often critiqued for impeding generalisability of findings due to social desirability bias exhibited by respondents [82] and experimental manipulations that may not be applicable in real-world settings [83]. In the present study, Reddit data offers the distinct advantage in obtaining candid qualitative responses [84,85] which stands out as a strength compared to previous studies that are primarily reliant on questionnaires [14]. Reddit is also uniquely responsive to topic modelling, given that all its posts are publicaly available unlike other social media platforms such as Facebook and Instagram [64]. The abundance of social media data thus provides a valuable chance for researchers to address research inquiries in innovative manners.

### 7.2 Parental gender differences in HP

The result of analysis consistently revealed that the terms ‘mother’ and ‘mom’ were most frequently used in the data. This is in line with studies that have found mothers engage in more HP than fathers from the perspectives of parents [[86], [87], [88]], children [22,86], and college administrators [89]. Notably, this study provides evidence that in conditions where users are not restricted by the methodological choice of researchers, spontaneous responses revealed predominantly maternal models of HP. This suggests that HP is present among fathers albeit to a much lesser degree than mothers. Given HP manifests itself differently for mothers and fathers, there is merit in further examining gender differences in HP.

### 7.3 Parenting during the COVID-19 pandemic

The results also showed a rapid increase in the number of subscribers of the subreddit r/helicopterparents since late 2019. In January 2020, the World Health Organization (WHO) declared COVID-19 a pandemic [90]. The impact of COVID-19 on parenting stress has negatively impacted parent-child relationships [91]. The series of uptrends in the subscriber count is parallel to parents working from home. This may be an indicator that HP is burgeoning in times of COVID-19. Research has also shown more salient effects of HP among students living with their parents as compared to those living away from their parents [92]. Therefore, when conducting research during or in the aftermath of the COVD-19 pandemic, it is important to consider how the pandemic has affected different contents around the respondents, especially family relationships and its potential long-term effects.

### 7.4 Mindfulness as a potential solution

The present study offers the potential to tailor meaningful interventions for HP that are directly relevant to users’ lived experiences. Pactitioners can provide parent-focused prevention and interventions that emphasise the importance of parental autonomy support in mitigating the ill effects of HP, [13]. Furthermore, the prominence of HP among mothers and the COVID-19 pandemic can become important targets for intervention. The effectiveness of mindfulness interventions has been extensively studied with parenting [[93], [94], [95], [96]], the pandemic [97,98], and emerging adults [99]. Thus, mindfulness training poses a viable solution to the problems of HP.

## 8 Limitatios and future directions

While this study is the first to provide insights from social data about HP, there are two notable limitations. First, the sociodemographic information of Reddit users is not publicly available [84]. Hence caution must be exercised when generalising the results of this study. Perhaps, creating a subreddit specific to an age group (e.g., emerging adulthood), gender, or geographic location may provide additional context to the phenomenon of HP. Further, information on the users and their parents’ social, cognitive, emotional, and mental functioning is warranted to fully understand the normativeness of the research population. This may appraise the generalisability of findings obtained from online forum conversations.

Second, not all topics are identified by the LDA modeling since responses to the original Reddit posts were not included in the corpus. Comments for each Reddit post may possess broad and varied discussions on the main topic [100]. These threads may contain information that is sensitive or less commonly experienced [64]. Thus, it is recommended that future research analyse Reddit posts and comments in tandem to increase the potential of topic extraction while new findings add to the existing literature on HP. Last, using a traditional approach of human coding may have provided more context to the findings given that the computer-based approach of topic modelling is developed solely based on a cluster of words. Although recent research suggests that both methods are comparable [101], more work is required to ascertain the viability of natural language processing techniques in studying psychological research.

## 9 Conclusion

This research adopted a novel, Big Data approach to understanding Reddit users’ experience with HP, contributing to the emerging efforts to amalgamate the distinct fields of psychology and computational linguistic research. Results revealed common environmental contexts of HP (i.e., school, college, work, and home) and its implication on college decisions, privacy, and social relationships. Our findings emphasised the lack of autonomy that HP fosters and encourages enhanced family communication patterns and mindfulness interventions. Furthermore, as with past research, our exploratory study supports more maternal than paternal models of HP, warranting further examination of gender in behavioural manifestations of HP. Considering the COVID-19 pandemic, future research must study the implications of high levels of parent-child contact on HP. Overall, these findings offer meaningful insights into HP through first-hand accounts.

## Additional information

No additional information is available for this paper.

## Author note

We have no conflict of interest to disclose.

This publication is funded by the Internal Research Fund of James Cook University, Singapore (HDRCF202312).

We express sincere gratitude to Mr R Sashitharan for his guidance in programming.

## CRediT authorship contribution statement

**Keerthigha C:** Conceptualization, Data curation, Formal analysis, Investigation, Methodology, Project administration, Resources, Software, Visualization, Writing – original draft, Writing – review & editing. **Smita Singh:** Conceptualization, Supervision, Writing – review & editing. **Kai Qin Chan:** Conceptualization, Supervision. **Nerina Caltabiano:** Supervision.

## Declaration of competing interest

The authors declare that they have no known competing financial interests or personal relationships that could have appeared to influence the work reported in this paper.

## Figures and Tables

**Table:** 

Recommended Ethical Code of Conduct [56,57]	Explanation of Compliance in Present Study
Web scraping must not be performed on websites that require registration (free or not) to view content.	Reddit is a free-to-access website, requiring no registration to view its content.
If access to data is restricted via technological means, researchers should not attempt to circumvent these technological restrictions and instead seek express consent from the website owner.	The website has no restrictions on data.
The approach of web scraping should be theory-driven rather than data-driven in the context of social science.	Research questions were formulated.
Ensure web scraping is not explicitly prohibited by the website's “terms of use” policy.	Web scraping is not explicitly or implicitly prohibited by the website's “terms of use”.
Ensure the website's data is not explicitly copyrighted.	The threads were user-generated and not from any copyrighted sources.
Ensure the research project does not encompass illegal or fraudulent use of the data.	The data was not illegally or fraudulently used.
Ensure web scraping does not possess the threat of causing material damage to the website or web server hosting the website.	*Pause codes* were input to prevent overwhelming the server. Pause codes restrict the speed at which the data is extracted. It pings the server once in 2 s, which is considered a safe ping frequency that will not overwhelm server capacity [102].
Ensure data collected from the website does not compromise individuals' privacy.	Users do not explicitly provide consent for their data to be mined. Thus, extra caution was taken when collecting and analysing their data to ensure they remain anonymous and unidentifiable.
Ensure data collected from the website does not reveal confidential information.	Data was not confidential.
Ensure research requiring the data does not possess the threat of reducing the value of the service provided by the website.	The data is publicly available, with or without the proposed data extraction.

## References

[1] Harlow L.L., Oswald F.L. (2016). Big data in psychology: introduction to the special issue. Psychol. Methods.

[2] Marengo D., Poletti I., Settanni M. (2020). The interplay between neuroticism, extraversion, and social media addiction in young adult Facebook users: testing the mediating role of online activity using objective data. Addict. Behav..

[3] Statista (2021). Reddit - statistics & facts. https://www.statista.com/topics/5672/reddit/#topicHeader__wrapper.

[4] Proferes N., Jones N., Gilbert S., Fiesler C., Zimmer M. (2021). Studying reddit: a systematic overview of disciplines, approaches, methods, and ethics. Social Media + Soc..

[5] Cullaty B. (2011). The role of parental involvement in the autonomy development of traditional-age college students. J. Coll. Student Dev..

[6] LeMoyne T., Buchanan T. (2011). Does “hovering” matter? Helicopter parenting and its effect on well-being. Socio. Spectr..

[7] Segrin C., Woszidlo A., Givertz M., Bauer A., Taylor Murphy M. (2012). The association between overparenting, parent‐child communication, and entitlement and adaptive traits in adult children. Fam. Relat..

[8] Winner N.A., Nicholson B.C. (2018). Overparenting and narcissism in young adults: the mediating role of psychological control. J. Child Fam. Stud..

[9] Padilla-Walker L.M., Nelson L.J. (2012). Black hawk down?: establishing helicopter parenting as a distinct construct from other forms of parental control during emerging adulthood. J. Adolesc..

[10] Locke J., Kavanagh D., Campbell M. (2016). Overparenting and homework: the student’s task, but everyone’s responsibility. J. Psychol. Couns. Sch..

[11] Rousseau S., Scharf M. (2018). Why people helicopter parent? An actor–partner interdependence study of maternal and paternal prevention/promotion focus and interpersonal/self-regret. J. Soc. Pers. Relat..

[12] Segrin C., Woszidlo A., Givertz M., Montgomery N. (2013). Parent and child traits associated with overparenting. J. Soc. Clin. Psychol..

[13] Hwang W., Jung E. (2022). Helicopter parenting versus autonomy supportive parenting? A latent class analysis of parenting among emerging adults and their psychological and relational well-being. Emerg. Adulthood.

[14] Kwon K.A., Yoo G., De Gagne J.C. (2017). Does culture matter? A qualitative inquiry of helicopter parenting in Korean American college students. J. Child Fam. Stud..

[15] Love H., Cui M., Allen J.W., Fincham F.D., May R.W. (2020). Helicopter parenting and female university students' anxiety: does parents' gender matter?. Families, Relat. Soc..

[16] Nelson L.J., Padilla-Walker L.M., McLean R.D. (2021). Longitudinal predictors of helicopter parenting in emerging adulthood. Emerg. Adulthood.

[17] Schiffrin H.H., Erchull M.J., Sendrick E., Yost J.C., Power V., Saldanha E.R. (2019). The effects of maternal and paternal helicopter parenting on the self-determination and well-being of emerging adults. J. Child Fam. Stud..

[18] Arnett J.J., Žukauskienė R., Sugimura K. (2014). The new life stage of emerging adulthood at ages 18–29 years: implications for mental health. Lancet Psychiatr..

[19] Darlow V., Norvilitis J.M., Schuetze P. (2017). The relationship between helicopter parenting and adjustment to college. J. Child Fam. Stud..

[20] Lenroot R.K., Giedd J.N. (2006). Brain development in children and adolescents: insights from anatomical magnetic resonance imaging. Neurosci. Biobehav. Rev..

[21] Kilford E.J., Garrett E., Blakemore S.J. (2016). The development of social cognition in adolescence: an integrated perspective. Neurosci. Biobehav. Rev..

[22] van Ingen D.J., Freiheit S.R., Steinfeldt J.A., Moore L.L., Wimer D.J., Knutt A.D., Roberts A. (2015). Helicopter parenting: the effect of an overbearing caregiving style on peer attachment and self‐efficacy. J. College Counselling.

[23] Kouros C.D., Pruitt M.M., Ekas N.V., Kiriaki R., Sunderland M. (2017). Helicopter parenting, autonomy support, and college students' mental health and well-being: the moderating role of sex and ethnicity. J. Child Fam. Stud..

[24] Schiffrin H.H., Liss M., Miles-McLean H., Geary K.A., Erchull M.J., Tashner T. (2014). Helping or hovering? The effects of helicopter parenting on college students' well-being. J. Child Fam. Stud..

[25] Willoughby B.J., Hersh J.N., Padilla-Walker L.M., Nelson L.J. (2015). “Back off”! Helicopter parenting and a retreat from marriage among emerging adults. J. Fam. Issues.

[26] Douce L.A., Keeling R.P. (2014). A strategic primer on college student mental health. https://tinyurl.com/94j48y5t.

[27] Yeo T.E.D. (2021). “Do you know how much I suffer?”: how young people negotiate the tellability of their mental health disruption in anonymous distress narratives on social media. Health Commun..

[28] Huh J., Ackerman M.S. (2012). Proceedings of the ACM 2012 Conference on Computer Supported Cooperative Work.

[29] Nabi R.L., Prestin A., So J. (2013). Facebook friends with (health) benefits? Exploring social network site use and perceptions of social support, stress, and well-being. Cyberpsychol., Behav. Soc. Netw..

[30] Kim Y., Kim B., Hwang H.S., Lee D. (2020). Social media and life satisfaction among college students: a moderated mediation model of SNS communication network heterogeneity and social self-efficacy on satisfaction with campus life. Soc. Sci. J..

[31] Munson S., Lauterbach D., Newman M., Resnick P., Morris M. (2010). Workshop on CSCW Research in Healthcare: Past, Present, and Future.

[32] Balani S., De Choudhury M. (2015). Proceedings of the 33rd Annual ACM Conference Extended Abstracts on Human Factors in Computing Systems.

[33] Berry N., Lobban F., Belousov M., Emsley R., Nenadic G., Bucci S. (2017). #WhyWeTweetMH: understanding why people use Twitter to discuss mental health problems. J. Med. Internet Res..

[34] Highton-Williamson E., Priebe S., Giacco D. (2015). Online social networking in people with psychosis: a systematic review. Int. J. Soc. Psychiatr..

[35] Choi D., Han J., Chung T., Ahn Y.Y., Chun B.G., Kwon T.T. (2015).

[36] He W., Zha S., Li L. (2013). Social media competitive analysis and text mining: a case study in the pizza industry. Int. J. Inf. Manag..

[37] Ingvaldsen J.E., Gulla J.A. (2012). Industrial application of semantic process mining. Enterprise Inf. Syst..

[38] Abdous M.H., He W. (2011). Using text mining to uncover students' technology‐related problems in live video streaming. Br. J. Educ. Technol..

[39] Hung J.L. (2012). Trends of e‐learning research from 2000 to 2008: use of text mining and bibliometrics. Br. J. Educ. Technol..

[40] Barbier G., Liu H. (2011). Social Network Data Analytics.

[41] Tadesse M.M., Lin H., Xu B., Yang L. (2019). Detection of depression-related posts in reddit social media forum. IEEE Access.

[42] Wan W., Sun J., Liu J., Yang S.W., Liu M., Xue J., Liu X. (2019). Using social media to explore the linguistic features in female adults with childhood sexual abuse by Linguistic Inquiry and Word Count. Hum. Behav. Emerging Technol..

[43] Coppersmith G., Leary R., Crutchley P., Fine A. (2018). Natural language processing of social media as screening for suicide risk. Biomed. Inf. Insights.

[44] Nanomi Arachchige I.A., Sandanapitchai P., Weerasinghe R. (2021). Investigating machine learning & natural language processing techniques applied for predicting depression disorder from online support forums: a systematic literature review. Information.

[45] Kang Y., Cai Z., Tan C.W., Huang Q., Liu H. (2020). Natural language processing (NLP) in management research: a literature review. J. Manag. Anal..

[46] Schwartz H.A., Eichstaedt J.C., Kern M.L., Dziurzynski L., Ramones S.M., Agrawal M., Ungar L.H. (2013). Personality, gender, and age in the language of social media: the open vocabulary approach. PLoS One.

[47] Grimmer J., Stewart B.M. (2013). Text as Data: the promise and pitfalls of automatic content analysis methods for political texts. Polit. Anal..

[48] Park G., Schwartz H.A., Eichstaedt J.C., Kern M.L., Kosinski M., Stillwell D.J., Seligman M.E. (2015). Automatic personality assessment through social media language. J. Pers. Soc. Psychol..

[49] Holtgraves T. (2011). Text messaging, personality, and the social context. J. Res. Pers..

[50] Odenweller K.G., Booth-Butterfield M., Weber K. (2014). Investigating helicopter parenting, family environments, and relational outcomes for millennials. Commun. Stud..

[51] Carr V.M., Francis A.P., Wieth M.B. (2021). The relationship between helicopter parenting and fear of negative evaluation in college students. J. Child Fam. Stud..

[52] Hayes K.N., Turner L.A. (2021). The relation of helicopter parenting to maladaptive perfectionism in emerging adults. J. Fam. Issues.

[53] Padilla-Walker L.M., Son D., Nelson L.J. (2021). Profiles of helicopter parenting, parental warmth, and psychological control during emerging adulthood. Emerg. Adulthood.

[54] Sattelberg W. (2021). *The demographics of reddit: who uses the site?* Alphr. https://tinyurl.com/j3c7cwzk.

[55] National Health and Medical Research Council (2018). https://tinyurl.com/feefvfzc.

[56] Landers R.N., Brusso R.C., Cavanaugh K.J., Collmus A.B. (2016). A primer on theory-driven web scraping: automatic extraction of big data from the Internet for use in psychological research. Psychol. Methods.

[57] Krotov V., Silva L. (2018). https://tinyurl.com/ymeyju6u.

[58] Boe B. (2021). https://praw.readthedocs.io/en/stable/.

[59] Meng M., Steinhardt S., Schubert A. (2018). Application programming interface documentation: what do software developers want?. J. Tech. Writ. Commun..

[60] Reddit (2021). https://www.reddit.com/dev/api/%20Rodrguez-Meirinhos.

[61] Loper E., Bird S. (2021). https://www.nltk.org/install.html.

[62] Blei D.M., Ng A.Y., Jordan M.I. (2003). Latent dirichlet allocation. J. Mach. Learn. Res..

[63] Bittermann A., Fischer A. (2018). How to identify hot topics in psychology using topic modeling. Z. für Psychol..

[64] Westrupp E.M., Greenwood C.J., Fuller-Tyszkiewicz M., Berkowitz T.S., Hagg L., Youssef G. (2022). Text mining of reddit posts: using latent Dirichlet allocation to identify common parenting issues. PLoS One.

[65] Subreddit Stats (2022). r/helicopterparents stats. https://subredditstats.com/r/helicopterparents.

[66] Chang J., Gerrish S., Wang C., Boyd-Graber J.L., Blei D.M. (2009). Reading tea leaves: how humans interpret topic models. Adv. Neural Inf. Process. Syst..

[67] Řehůřek R. (2021). https://radimrehurek.com/gensim/models/coherencemodel.html.

[68] Mabey B. (2015). https://pyldavis.readthedocs.io/en/latest/readme.html.

[69] Etkin R.G., Bowker J.C., Simms L.J. (2021). Friend overprotection in emerging adulthood: associations with autonomy support and psychosocial adjustment. J. Genet. Psychol..

[70] Kim S.Y., Wang Y., Orozco-Lapray D., Shen Y., Murtuza M. (2013). Does “tiger parenting” exist? Parenting profiles of Chinese Americans and adolescent developmental outcomes. Asian Am. J. Psychol..

[71] Segrin C., Givertz M., Swaitkowski P., Montgomery N. (2015). Overparenting is associated with child problems and a critical family environment. J. Child Fam. Stud..

[72] Barry C.M., Madsen S.D., DeGrace A., Arnett J.J. (2016). The Oxford Handbook of Emerging Adulthood.

[73] Hong J.C., Hwang M.Y., Kuo Y.C., Hsu W.Y. (2015). Parental monitoring and helicopter parenting relevant to vocational student's procrastination and self-regulated learning. Learn. Indiv Differ.

[74] Rote W.M., Olmo M., Feliscar L., Jambon M.M., Ball C.L., Smetana J.G. (2020). Helicopter parenting and perceived overcontrol by emerging adults: a family-level profile analysis. J. Child Fam. Stud..

[75] Gaertner A.E., Rathert J.L., Fite P.J., Vitulano M., Wynn P.T., Harber J. (2010). Sources of parental knowledge as moderators of the relation between parental psychological control and relational and physical/verbal aggression. J. Child Fam. Stud..

[76] Rodríguez-Meirinhos A., Vansteenkiste M., Soenens B., Oliva A., Brenning K., Antolín-Suárez L. (2020). When is parental monitoring effective? A person-centered analysis of the role of autonomy-supportive and psychologically controlling parenting in referred and non-referred adolescents. J. Youth Adolesc..

[77] Cutright M. (2008). From helicopter parent to valued partner: shaping the parental relationship for student success. N. Dir. High. Educ..

[78] Hammonds J.R. (2015). A model of privacy control: examining the criteria that predict emerging adults' likelihood to reveal private information to their parents. West. J. Commun..

[79] Son D., Padilla-Walker L.M. (2021). Longitudinal associations among perceived intrusive parental monitoring, adolescent internalization of values, and adolescent information management. J. Child Fam. Stud..

[80] Jiao J., Segrin C. (2021). Overparenting and emerging adults' insecure attachment with parents and romantic partners. Emerg. Adulthood.

[81] Cook E.C. (2020). Understanding the associations between helicopter parenting and emerging adults' adjustment. J. Child Fam. Stud..

[82] Krumpal I. (2013). Determinants of social desirability bias in sensitive surveys: a literature review. Qual. Quantity.

[83] Innes J.M., Morrison W., B (2021). Experimental studies of human–robot interaction: threats to valid interpretation from methodological constraints associated with experimental manipulations. Int. J. Soc. Robot..

[84] Amaya A., Bach R., Keusch F., Kreuter F. (2021). New data sources in social science research: things to know before working with reddit data. Soc. Sci. Comput. Rev..

[85] Jamnik M.R., Lane D.J. (2017). The use of Reddit as an inexpensive source for high- quality data. Practical Assess. Res. Eval..

[86] Fingerman K.L., Cheng Y.-P., Wesselmann E.D., Zarit S., Fustenberg F., Birditt K.S. (2012). Helicopter parents and landing pad kids: intense parental support of grown children. J. Marriage Fam..

[87] Rousseau S., Scharf M. (2015). “I will guide you”: the indirect link between overparenting and young adults' adjustment. Psychiatr. Res..

[88] Scharf M., Rousseau S., Bsoul S. (2017). Overparenting and young adults' interpersonal sensitivity: cultural and parental gender-related diversity. J. Child Fam. Stud..

[89] Somers P., Settle J. (2010). The helicopter parent: research toward a typology. Coll. Univ.:J. Am. Assoc. Colleg. Registrars.

[90] WHO (2020).

[91] Chung G., Lanier P., Wong P.Y.J. (2020). Mediating effects of parental stress on harsh parenting and parent-child relationship during coronavirus (COVID-19) pandemic in Singapore. J. Fam. Violence.

[92] Hong P., Cui M. (2020). Helicopter parenting and college students' psychological maladjustment: the role of self-control and living arrangement. J. Child Fam. Stud..

[93] Burgdorf V., Szabó M., Abbott M.J. (2019). The effect of mindfulness interventions for parents on parenting stress and youth psychological outcomes: a systematic review and meta-analysis. Front. Psychol..

[94] Kil H., Antonacci R., Shukla S., De Luca A. (2021). Mindfulness and parenting: a meta-analysis and an exploratory meta-mediation. Mindfulness.

[95] Chaplin T.M., Turpyn C.C., Fischer S., Martelli A.M., Ross C.E., Leichtweis R.N., Sinha R. (2021). Parenting-focused mindfulness intervention reduces stress and improves parenting in highly stressed mothers of adolescents. Mindfulness.

[96] Coatsworth J.D., Duncan L.G., Nix R.L., Greenberg M.T., Gayles J.G., Bamberger K.T., Demi M.A. (2015). Integrating mindfulness with parent training: effects of the mindfulness-enhanced strengthening families program. Dev. Psychol..

[97] Bossi F., Zaninotto F., D'Arcangelo S., Lattanzi N., Malizia A.P., Ricciardi E. (2022). Mindfulness-based online intervention increases well-being and decreases stress after covid-19 lockdown. Sci. Rep..

[98] Yeun Y.R., Kim S.D. (2022). Psychological effects of online-based mindfulness programs during the COVID-19 pandemic: a systematic review of randomized controlled trials. Int. J. Environ. Res. Publ. Health.

[99] Duprey E.B., McKee L.G., O'Neal C.W., Algoe S.B. (2018). Stressful life events and internalizing symptoms in emerging adults: the roles of mindfulness and gratitude. Mental Health & Prevent..

[100] Weninger T. (2014). An exploration of submissions and discussions in social news: mining collective intelligence of reddit. Soc. Netw. Anal. Mining.

[101] Nanda G., Jaiswal A., Castellanos H., Zhou Y., Choi A., Magana A.J. (2023). Evaluating the coverage and depth of latent dirichlet allocation topic model in comparison with human coding of qualitative data: the case of education research. Mach. Learn. Knowl. Extract..

[102] Larson W. (2008). https://lethain.com/an-introduction-to-compassionate-screenscraping/.

